# Rutin alleviated lipopolysaccharide-induced damage in goat rumen epithelial cells

**DOI:** 10.5713/ab.23.0028

**Published:** 2023-10-27

**Authors:** Jinshun Zhan, Zhiyong Gu, Haibo Wang, Yuhang Liu, Yanping Wu, Junhong Huo

**Affiliations:** 1 Institute of Animal Husbandry and Veterinary, Jiangxi Academy of Agricultural Science, Nanchang 330200, China; 2 College of Animal Science and Veterinary Medicine, Tianjin Agricultural University, Tianjin 300384, China; 3 College of Animal Science and Technology, Gansu Agricultural University, Lanzhou 730070, China

**Keywords:** Antioxidant Property, Anti-inflammatory Activity, Cell Apoptosis, Goat Rumen Epithelial Cells, Lipopolysaccharide, Rutin

## Abstract

**Objective:**

Rutin, also called vitamin P, is a flavonoids from plants. Previous studies have indicated that rutin can alleviate the injury of tissues and cells by inhibiting oxidative stress and ameliorating inflammation. There is no report on the protective effects of rutin on goat rumen epithelial cells (GRECs) at present. Hence, we investigated whether rutin can alleviate lipopolysaccharide (LPS)-induced damage in GRECs.

**Methods:**

GRECs were cultured in basal medium or basal medium containing 1 μg/mL LPS, or 1 μg/mL LPS and 20 μg/mL rutin. Six replicates were performed for each group. After 3-h culture, the GRECs were harvested to detect the relevant parameters.

**Results:**

Rutin significantly enhanced the cell activity (p<0.05) and transepithelial electrical resistance (TEER) (p<0.01) and significantly reduced the apoptosis rate (p<0.05) of LPS-induced GRECs. Rutin significantly increased superoxide dismutase, glutathione peroxidase, and catalase activity (p<0.01) and significantly decreased lactate dehydrogenase activity and reactive oxygen species and malondialdehyde (MDA) levels in LPS-induced GRECs (p<0.01). The mRNA and protein levels of interleukin 6 (IL-6), IL-1β, and C-X-C motif chemokine ligand 8 (CXCL8) and the mRNA level of tumor necrosis factor-α (TNF-α) and chemokine C-C motif ligand 5 (CCL5) were significantly increased in LPS-induced GRECs (p<0.05 or p<0.01), while rutin supplementation significantly decreased the mRNA and protein levels of IL-6, TNF-α, and CXCL8 in LPS-induced GRECs (p<0.05 or p<0.01). The mRNA level of toll-like receptor 2 (TLR2), and the mRNA and protein levels of TLR4 and nuclear factor κB (NF-κB) was significantly improved in LPS-induced GRECs (p<0.05 or p<0.01), whereas rutin supplementation could significantly reduce the mRNA and protein levels of TLR4 (p<0.05 or p<0.01). In addition, rutin had a tendency of decreasing the protein levels of CXCL6, NF-κB, and inhibitor of nuclear factor kappa-B alpha (0.05< p<0.10). Rutin could significantly decreased interferon regulatory factor 3 mRNA expression in LPS-induced GRECs (p<0.05), whereas interferon induced protein with tetratricopeptide repeats 3 (IFIT3) and toll-interacting protein (TOLLIP) mRNA expression was not significantly different between the groups. LPS reduced the tight junction protein zonula occludin 1 (ZO-1) level in GRECs whereas rutin enhanced it. Rutin significantly improved tight junction protein *Claudin-1* mRNA expression in LPS-induced GRECs (p<0.01), but could not affect tight junction protein *Occludin* mRNA expression.

**Conclusion:**

Rutin alleviated LPS-induced barrier damage in GRECs by improving oxidation resistance and anti-inflammatory activity, which may be related to TLR/NF-κB signaling pathway inhibition.

## INTRODUCTION

The rumen epithelium plays a vital role in facilitating nutrient absorption and metabolization in ruminants, with its health status directly impacting the health of ruminants. The rumen epithelial barrier is composed of a multi-cellular structure and intercellular linking complex, which effectively prevents the translocation of lipopolysaccharide (LPS) into the bloodstream. A high-concentrate diet can induce ruminal acidosis, leading to the release of LPS, which are cell wall components of gram-negative bacteria in the rumen. A high concentration of LPS in the rumen can impair the structural integrity of the rumen epithelium and ruminal epithelial barrier function, leading to translocation into bloodstream and triggering an inflammatory response in ruminant organisms [1]. Cells possess an autonomous and orderly self-control metabolic system, which can yield a plethora of experimental materials. Cell culture enables direct observation of the morphological structure and vital activities of cells, making it a widely utilized tool in various disciplines such as cytology, genetics, immunology, experimental medicine and oncology. Cell culture offers a cost-effective alternative to the study of large animals (dairy cow, sheep, *etc*). Previous study found cultured ruminal epithelial cells respond to LPS stimulation by increasing transcription of pro-inflammatory genes and this transcriptional response was influenced by the dose, duration, and frequency of LPS exposure [2]. This establishes a basis for investigating the impact of acidosis on rumen epithelial function at a mechanistic level.

Antibiotics, such as monensin, play an important role in preventing rumen acidosis in ruminants. However, the large amounts of antibiotics used in ruminant fodder generate bacterial drug resistance and drug residues in animal products that can present a serious threat to human health. Therefore, there is an urgent need to identify substitutions for the current antibiotics. A moderate level of flavonoids increased growth performance and decreased the risk of ruminal acidosis in cattle on high-concentrate diet, which have the similar effects of monensin [3]. Hence, antibiotics may be replaced by flavonoids in ruminant production. Rutin, or rutoside, quercetin-3-*O*-rutinoside, or vitamin P, is a natural flavonoid glycoside that is a secondary metabolite in plants, which has antioxidant, anti-hypertension, anti-diabetes, anti-inflammation, and cardioprotective functions. Previously, we reported that rutin promoted the growth performance of sheep by enhancing antioxidant capacity and rumen fermentation and regulating hormone secretion, and improved meat quality of goats [4–6]. These studies indicate that rutin might be used in ruminant production as a feed additive. Its absorption and metabolism is the premise of the biological functions of rutin. Rutin is stable in gastric and small intestinal fluid for 2 to 4 h and can be absorbed by the gastrointestinal tract. Guo [7] reported that the peak plasma concentration of rutin and quercetin in dairy cows was at 1 h and 8 h after intake, respectively. Previous studies found rutin could relieve ethanol-induced gastric lesions and alleviate ischemia-reperfusion-induced injury of rat small intestine, respectively [8,9]. In addition, rutin has higher bioavailability in ruminants compared to monogastric species. Hence, rutin can be absorbed and metabolized in the gastrointestinal tract, then play a role in protecting the rumen and intestine of ruminants.

According to the study of Shi et al [10], an increase in LPS concentration and incubation time resulted in a significant reduction of the relative growth rate of cell. LPS can decrease the activities of antioxidant enzymes glutathione peroxidase (GSH-PX), catalase (CAT) and superoxide dismutase (SOD), elevate the malondialdehyde (MDA) content, concentrations of pro-inflammatory cytokines interleukin-1 (IL-1) and IL-6 as well as nitric oxide (NO), and induce the expression of iNOS in bovine mammary epithelial cells. In addition, LPS-induced primary goat mammary epithelial cells for 3 h led to an increase in expression of C-C motif chemokine ligand 2 (CCL 2), C-X-C motif chemokine ligand 6 (CXCL 6), IL-6, CXCL 8, prostaglandin-endoperoxide synthase 2 (PTGS 2), interferon induced protein with tetratricopeptide repeats 3 (IFIT 3), myeloid differentiation primary response gene 88 (MyD88), nuclear factor of kappa light polypeptide gene enhancer in B-cells 1 (NFKB1) and toll-like receptor 4 (TLR 4) [11]. Our previous study revealed a significant decrease in the activities of GSH-PX, CAT, and SOD, as well as a significant elevation in the relative mRNA transcript levels of cytokines (CCL 5, CXCL 6, CXCL 8, IL-6, tumor necrosis factor-α [TNF-α], and IL-1β) and regulatory factors (TLR 2, nuclear factor κB [NF-κB], and MyD88) in LPS-induced goat rumen epithelial cells (GRECs) [12]. These studies have demonstrated that LPS can elicit proinflammatory effects in cells by inducing oxidative stress. Previous studies found rutin can attenuate LPS-induced inflammation in muscle cells and apoptosis in human bronchial epithelial cell line cells by inhibiting oxidative stress [13,14]. It showed that rutin has the role of protecting cells. Quercetin (aglycones of rutin) could alleviate LPS-induced GRECs damage by enhancing antioxidant capacity and inhibiting inflammatory response [12]. At present, there has been no report on the protective effect of rutin against LPS-induced injury in GRECs. To verify whether rutin can protect GRECs like as quercetin, we examined its effects on the oxidation resistance, anti-inflammation, and barrier function of LPS-induced GRECs. This study was aimed at providing a theoretical basis for future studies on rutin protection of the ruminal epithelium *in vivo*.

## MATERIALS AND METHODS

This experiment was performed according to the guidelines approved by the Animal Ethics Committee of Animal Husbandry and Veterinary, Jiangxi Academy of Agricultural Sciences (2010-JAAS-XM-01).

### Cell culture and experimental design

The GRECs obtained from Boer goat ruminal epithelium were from the Institute of Animal Culture Collection and Application (IACCA), Yangzhou University. The GRECs (1×10^5^ cells/mL) were suspended with basal medium (Dulbecco’s modified Eagle’s medium [DMEM]/F12 medium), inoculated into culture plates, and cultured in an incubator at 37°C with 5% CO_2_ saturated humidity until they had completely adhered to the plates. The GRECs were subcultured until the third passage. When the adherence of GRECs reached 80% to 90%, the GRECs were digested with trypsin and washed twice with phosphate-buffered saline (PBS). Then, the GRECs were divided into three groups and cultured in basal medium without rutin or LPS (control group, Con) or basal medium containing 1 μg/mL LPS (L), or 1 μg/mL LPS and 20 μg/mL rutin (L+R). Six replicates were performed for each group. The basal medium was supplemented with rutin (≥98% purity, Yuanye Bio-Technology, Shanghai, China) dissolved with dimethyl sulfoxide (DMSO, Cell level; Sigma-Aldrich, St. Louis, MO, USA). All experimental groups received 0.1% DMSO. After 3-h culture, the GRECs were harvested to detect the relevant parameters.

### Cell proliferative activity

GREC proliferative activity was detected with Cell Counting Kit-8 (Beyotime Biotechnology, Shanghai, China). The GRECs (1×10^4^ cells per well) were inoculated into 96-well plates and incubated at 37°C with 5% CO_2_ saturated humidity for 3 h, then incubated for 3 h with 10 μg/mL CCK-8 solution. Then, the absorbance of the GRECs was assayed at a wavelength of 450 nm using a microplate spectrophotometer (Multiskan Spectrum; Thermo Fisher Scientific, Waltham, MA, USA). The cell viability was calculated as follows:


$$\frac{\text{At}-\text{Ab}}{\text{Ac}-\text{Ab}}\times 100\%,$$

where At, Ac, and Ab are the absorbances of the treatment groups, control group, and blank group (containing only basal medium), respectively.

### Determination of transepithelial electrical resistance

The GRECs (1×10^5^ cells per cm^2^) were seeded into a transwell chamber (Costar, Coring Inc, New York, NY, USA) with 4.5 μm pores fit-ted into a 6-well plate. The other one transwell was used as a blank, which did not inoculate the GRECs. Transepithelial electrical resistance (TEER) of all treatment groups was measured by an epithelial volt-ohm meter with a chopstick electrode (Milli-cell ERS-2; EMD Millipore, Billerica, MA, USA) after finishing incubation of GRECs. The electrode was inserted the basolateral chamber and the apical chamber, respectively. Each monolayer was detected three times, and paid attention to preventing electrode contact with the monolayer. The mean resistance of a blank was deducted from all treatment groups. Finally, the unit area resistance was calculated by dividing resistance values by effective membrane area (4.5 cm^2^).

### Apoptosis assay

GREC apoptosis was assayed by an annexin V–propidium iodide (PI) apoptosis detection kit (Absin, Shanghai, China). The GRECs (1×10^5^ cells/mL) were seeded in 6-well plates, cultured in the treatment media for 3 h, detached with trypsin, then collected after washing twice with cold PBS. The GRECs were suspended with tag solution containing annexin V and PI, then incubated in the dark for 15 min at room temperature. The subsequent steps were performed according to the kit instructions. Finally, the cell fluorescence was detected using a flow cytometer (CytoFLEX; Beckman Coulter, Brea, CA, USA). The wavelength of exciting light is 488 nm. Fluorescein isothiocyanate fluorescence was detected by a passband filter with a wavelength of 515 nm and PI was detected by filter with wavelength greater than 560 nm. The left bottom of quadrant shows living cells, the upper right of quadrant shows necrosis cells, and the right bottom of quadrant shows apoptosis cells on the scatter plot of the bivariable flow cytometry.

### Anti-oxidative parameters

Cell suspensions (2 mL) containing 1×10^5^ GRECs/mL were inoculated into 12-well plates. The basal medium was replaced by the treatment medium after cell attachment. The GRECs were incubated for 3 h in an incubator and then collected for detecting SOD, GSH-PX, CAT, and lactate dehydrogenase (LDH) activity and MDA concentrations according to the kit instructions. The anti-oxidative parameter detection kits were all from the Nanjing Jiancheng Bioengineering Institute (Nanjing, China).

Reactive oxygen species (ROS) were detected as follows: GRECs cultured in treatment medium for 3 h were harvested and incubated for 1 h in basal medium containing 2,7-dichlorodihydrofluorescein diacetate (DCFH-DA). Subsequently, the GRECs were trypsinized and washed twice with PBS before resuspension with 300 μL PBS. The fluorescence of the GRECs was measured by flow cytometry. The excited and emitted wavelength of flow cytometry is 488 nm and 525 nm, respectively.

### Immunofluorescence assay of tight junction protein zonula occludin 1

After incubation in the treatment medium, the GRECs were fixed in 4% paraformaldehyde for 30 min, washed thrice with PBS, and sealed with 3% horse serum at room temperature for 30 min. After the horse serum had been removed, the GRECs were incubated with primary antibody against zonula occludin 1 (ZO-1) (Abcam, Shanghai, China) overnight in a refrigerator at 4°C, then washed thrice with PBS. Next, the GRECs were incubated for 1 h in the dark at room temperature with the secondary antibody (Abcam, China) containing green fluorescence, then incubated in DAPI staining solution (Solarbio, Beijing, China) at room temperature for 5 min. Finally, the cells were washed three times in PBS and observed and visualized using a fluorescence microscope (Olympus, Tokyo, Japan).

### Real-time fluorescence quantitative polymerase chain reaction

Total RNA was extracted from the GRECs according to the instructions of an RNA extraction kit (Tiangen Biotech, Beijing, China). Reverse transcription was performed using a reverse transcription kit (Takara, Dalian, China). The reverse transcription was performed in a reaction mixture with a total volume of 20 μL containing 10 μL master mix, 1 μL PrimeScript RT Enzyme Mix I and RT Primer Mix, 4 μL 5× PrimeScript Buffer 2 and RNase-free dH_2_O. The reverse transcription reaction condition was 37°C for 15 min and 85°C for 5 s.

The real-time fluorescence quantitative polymerase chain reaction (qRT-PCR) was performed using a SYBR Premix Ex Taq II kit (Takara, China). The qRT-PCR primers were synthesized by Invitrogen (Shanghai, China); Table 1 lists their sequences. The primer sequences of genes excepting *Claudin-1* and *Occludin* in Table 1 were from the thesis of Hu et al [15]. The qRT-PCR was performed in a total volume of 20 μL containing Premix Ex Taq II (10 μL), forward primer (0.8 μL), reverse primer (0.8 μL), complementary DNA (cDNA) template (2 μL), and PCR-grade water (6.4 μL). The qRT-PCR was performed on a LightCycler 96 real-time PCR instrument (Roche, Basel, Switzerland) under the following conditions: initial denaturation at 95°C for 30 s, followed by 40 cycles of PCR at 95°C for 5 s and 60°C for 30 s. Glyceraldehyde-3-phosphate dehydrogenase (*GAPDH*) was used as the reference gene for calculating the relative expression of the target genes with the comparative threshold cycle (2^−ΔΔCt^) method.

### Determination of protein content by enzyme-linked immunosorbent assay

First, the supernatant of the GRECs was collected and used for measuring the protein contents of cytokines (IL-1β, IL-6, and TNF-α) and chemokines (CXCL6 and CXCL8). Subsequently, the GRECs were collected for extracting protein. The proteins extracted from GRECs were used for detecting the protein contents of key molecules in TLR/NF-κB signal pathway, such as toll-like receptor 2 (TLR2), TLR4, NF-κB, and inhibitor of nuclear factor kappa-B alpha (IκBα). The method of protein extraction was as follow. The GRECs were added 1 mL extracting solution and crushed by ultrasonic wave (ice bath, 200 W, ultrasonic for 3 s, 10 s for interval, repeat 30 times). Then, the supernatant was collected by centrifuging at 4°C, 12,000 rpm for 10 min after finishing the ultra sonication. The protein content of supernatant was detected according to the instruction of BCA protein assay kit (Solarbio, China). Finally, the contents of those proteins were detected according to the manufacturer’s instructions of the enzyme-linked immunosorbent assay (ELISA) kits (FANKEW; Shanghai, China). The ELISA kits of IL-1β (F6400-B; FANKEW, China), IL-6 (F3885-B; FANKEW, China), TNF-α (F3849-B; FANKEW, China), CXCL6 (F7100187-B; FANKEW, China), CXCL8 (F7100172-B; FANKEW, China), TLR2 (F7100169-B; FANKEW, China), TLR4 (F7100176-B; FANKEW, China), NF-κB (F7100181-B; FANKEW, China) and IκBα (F7100194-B; FANKEW, China) were bought from Shanghai Kexing Trading Co., Ltd (Shanghai, China).

### Statistical analysis

Data were pretreated using Excel in WPS Office 2019 (Kingsoft, Beijing, China) and analyzed using one-way analysis of variance in SPSS 21.0 (IBM, Chicago, IL, USA). A Duncan multiple comparison test was performed and the data are reported as the means and standard errors of the mean (SEM). Statistically significant differences between treatment groups were determined at p<0.05, while extremely significant differences were observed at p<0.01. 0.05<p<0.10 was taken as an indication of tendency.

## RESULTS

### Effects of rutin on the cell activity of LPS-induced GRECs

**Figure 1:**
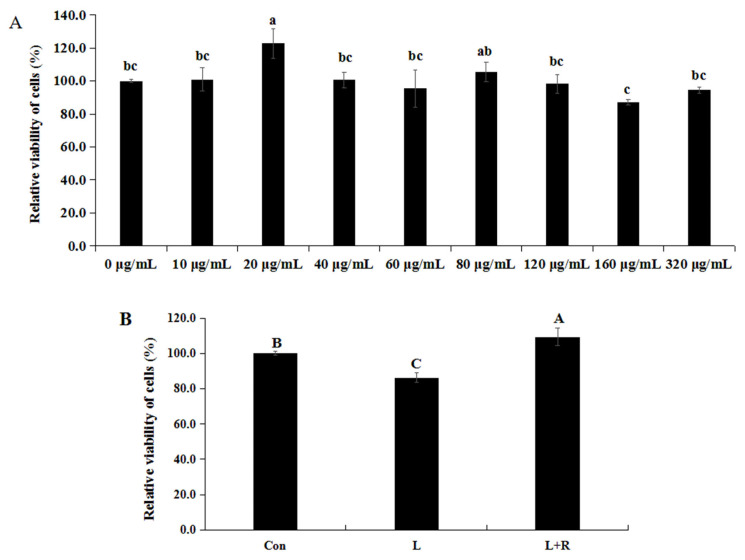
Effect of different levels of rutin on the viability of GRECs (A). Effect of rutin on the activity of LPS-induced GRECs (B). The graph depicts the statistical results on GREC relative viability. Data are the means±standard errors of the mean. LPS, lipopolysaccharide; GRECs, goat rumen epithelial cells; Con, control group; L, LPS group; L+R, LPS+rutin group. Different lowercase letters (p<0.05) and capital letters (p<0.01) on the columns indicate significant differences.

Effects of different concentrations of rutin on the cell activity of GRECs are showed in Figure 1A. The cell activity of GRECs cultured in the medium supplemented 20 μg/mL of rutin was higher than that in other groups except the 80 μg/mL of rutin (p<0.05). Thus, we chose 20 μg/mL as the concentration of rutin in the following experiment. Effects of rutin on the cell activity of LPS-induced GRECs are exhibited in Figure 1B. The cell activity of GRECs was significantly reduced in the L group compared to the other groups (p<0.01) and was highest in the L+R group (p<0.01). Those results indicated that rutin had the function of improving cell viability of LPS-induced GRECs.

### Effects of rutin on the permeability of LPS-induced GRECs

**Figure 2:**
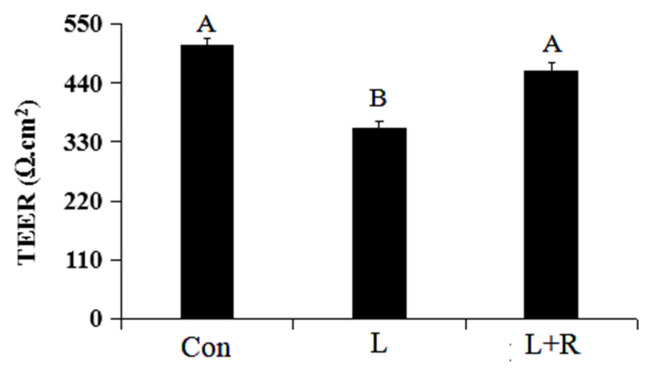
Effects of rutin on the permeability of LPS-induced GRECs. The graph depicts the statistical results on the permeability of GRECs. Data are the means±standard errors of the mean. LPS, lipopolysaccharide; GRECs, goat rumen epithelial cells; Con, control group; L, LPS group; L+R, LPS+rutin group. Columns with different capital letters indicate significant differences (p<0.01).

The results of the permeability of GRECs are displayed in Figure 2. Compared to the control group, the TEER of GRECs in the L group was significantly reduced (p<0.01). Meanwhile, the rutin can significantly enhance the TEER of GRECs induced by LPS (p<0.01). The results indicated that rutin can increase the TEER and decrease the the permeability of GRECs.

### Effects of rutin on the apoptosis of LPS-induced GRECs

**Figure 3:**
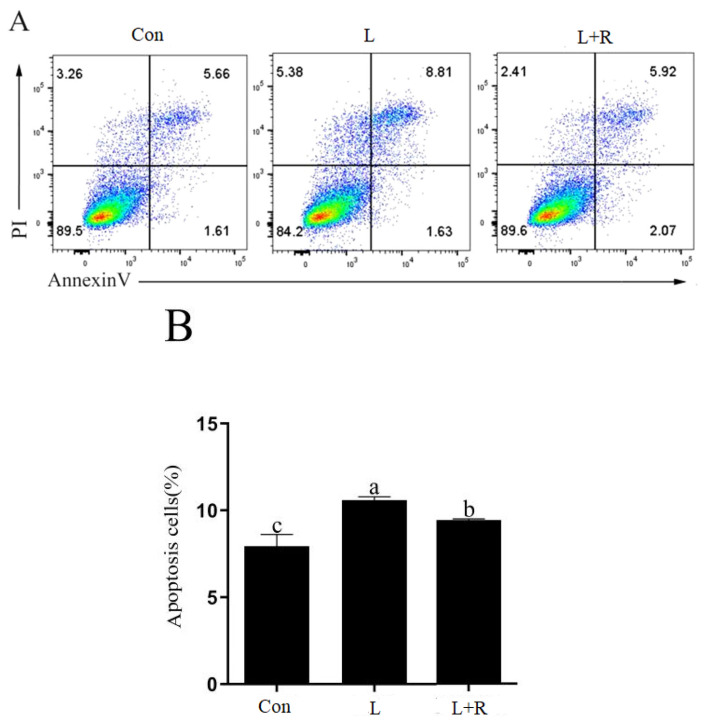
Effects of rutin on the apoptosis of LPS-induced GRECs. (A) The apoptotic rate was detected by flow cytometry. (B) The statistical result of the apoptotic rate in GRECs. Data are the means±standard errors of the mean. LPS, lipopolysaccharide; GRECs, goat rumen epithelial cells; Con, control group; L, LPS group; L+R, LPS+rutin group. Columns with different lowercase letters indicate significant differences (p<0.05).

The apoptosis of GRECs in different treatment groups are showed in Figure 3A and 3B. Compared to the control, apoptosis was significantly increased in the L and L+R groups (p<0.05). Apoptosis in the L+R group was significantly lower than that in the L group (p<0.05). The results showed rutin could inhibit apoptosis of GRECs induced by LPS.

### Effects of rutin on the antioxidative parameters of LPS-induced GRECs

The results for the GREC antioxidative parameters are depicted in Table 2. Compared with the control, SOD, GSH-PX, and CAT activity in the L group was significantly decreased, but LDH activity and ROS and MDA levels were significantly increased (p<0.01). Compared to the L group, SOD, GSH-PX, and CAT activity in the L+R group was significantly enhanced but LDH activity and ROS and MDA levels were significantly reduced (p<0.01). The results suggested that rutin supplementation enhances the antioxidant capacity of LPS-induced GRECs.

### Effects of rutin on relative expression of cytokine genes in LPS-induced GRECs

The mRNA relative expression of cytokine genes in different treatment groups are displayed in Table 3. Compared to the control, the mRNA relative expression of IL-6 (p<0.05), TNF-α (p<0.01), and IL-1β (p<0.01) was enhanced significantly in the L group. Rutin significantly reduced the mRNA relative expression of TNF-α in the LPS-induced GRECs (p<0.01), whereas it had no significant effect on IL-6 and IL-1β mRNA relative expression (p>0.05). Compared to the control group, CCL5, CXCL6, and CXCL8 mRNA relative expression was significantly increased in the L and L+R groups (p<0.01). CXCL6 and CXCL8 mRNA relative expression was significantly lower in the L+R group than in the L group (p<0.01). The results indicated that rutin might inhibit the inflammatory reaction of GRECs induced by LPS.

### Effects of rutin on the relative expression of regulatory factor genes in LPS-induced GRECs

The mRNA relative expression of regulatory factor genes in GRECs from different treatment groups are displayed in Table 4. Compared to the control, TLR2 (p<0.01), TLR4 (p<0.05), and NF-κB (p<0.01) mRNA relative expression was significantly increased in the L. Compared to the L group, TLR2 (p<0.01), TLR4 (p<0.05), NF-κB (p<0.01) and interferon (IFN) regulatory factor 3 (IRF3) (p<0.05) mRNA relative expression was significantly decreased in the L+R group. *MyD88* mRNA relative expression in the L+R group was significantly higher than that in the the control (p<0.05), whereas interferon induced protein with tetratricopeptide repeats 3 (IFIT3) and toll-interacting protein (TOLLIP) mRNA relative expression was not significantly different between the groups. The results showed rutin might suppress the inflammatory reaction in LPS-induced GRECs by regulating the expression of *TLRs* and *NF-κB*.

### Effects of rutin on protein contents of cytokines in LPS-induced GRECs

The protein contents of cytokines in cells are showed in Table 5. Compared to the control, the protein contents of TLR4, NF-κB, IL-1β, and CXCL8 in the L group was significantly increased (p<0.05), whereas the protein content of TLR2 had no significant difference in all treatment groups (p<0.05). Compared to the L group, the protein contents of TLR4, IL-6, TNF-α, and CXCL8 in the L+R group was significantly decreased (p<0.05). The protein contents of NF-κB and CXCL6 had a decrease tendency in the L+R group compared with the L group (p = 0.06). Compared to the L group, the protein content of IκBα in the L+R group had an increasing tendency (p = 0.08). The results indicated that rutin might affect the inflammatory reaction in LPS-induced GRECs by regulating secretion of cytokines protein.

### Effects of rutin on tight junction protein expression in LPS-induced GRECs

**Figure 4:**
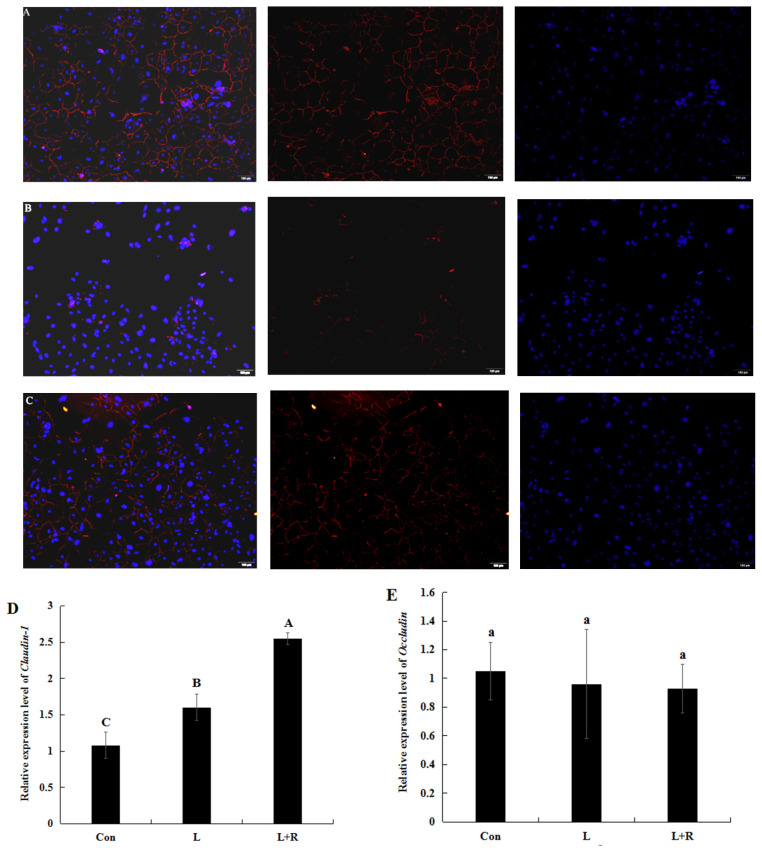
Effects of rutin on TJ proteins of LPS-induced GRECs. (A–C) Immunofluorescence assay of ZO-1 expression in the control group (A), L group (B), and L+R group (C). The red in the Figure A to C shows immunofluorescence localization of ZO-1 and blue shows the nucleus of GRECs. (D, E) The statistical results of the relative mRNA expression of Claudin-1 (D) and Occludin (E). Data are the means±standard errors of the mean. TJ, tight junction; LPS, lipopolysaccharide; GRECs, goat rumen epithelial cells; ZO-1, zonula occludin 1; Con, control group; L, LPS group; L+R, LPS+rutin group. Means with lowercase letters (p<0.05) and capital letters (p<0.01) on the columns are different.

The expression level of the tight junction (TJ) protein (ZO-1, Claudin-1, and Occludin) in the GRECs from different treatment groups are depicted in Figure 4A to 4E. The edge of the LPS-induced GRECs exhibited slight red spotty staining and fewer continuous grid structures (Figure 4B), whereas the edges of rutin-supplemented GRECs demonstrated continuous grid structure (Figure 4C). This indicated that rutin enhanced ZO-1 expression levels in the LPS-induced GRECs. Claudin-1 (*CLDN*) mRNA relative expression in the L+R group was significantly higher than that in the L group (p< 0.01), while it was lowest in the control (Figure 4D). Occludin (*OCLN*) mRNA relative expression (Figure 4E) had no significant difference in all groups (p<0.05). It illustrated that rutin might improve the barrier function of LPS-induced GRECs in this study.

## DISCUSSION

Shi et al [10] found that the relative growth rate of bovine mammary epithelial cells was significantly decreased with increasing the concentration of LPS and the incubation time. In addition, the cell viability of GRECs was reduced with increasing the LPS concentration in a certain range [16]. It showed LPS exerts a negative effect on cell proliferation. This study agreed with it. Previously, we found that quercetin supplementation could enhance the proliferation of LPS-induced GRECs [13]. The results of this study are consistent with it. However, according to the study of Kent-Dennis et al [2], LPS exposure did not negatively affect cell viability of bovine ruminal epithelial cells, which was different with us. The reason of it may be relation to different LPS concentration and treatment time. Apoptosis is a form of programmed cell death that plays a vital role in regulating cell development, cell aging, and the immune reactions of cells in tissues. Dai [17] reported that LPS induced by long-term high-concentrate diets induced rumen epithelial cell apoptosis *in vivo* and our results agree with this. The results of study by Paudel et al [14] showed rutin has the effects of potent anti-apoptotic activity against LPS induced damage. We also observed the similar results. In conclusion, rutin could promote the proliferation and inhibited the apoptosis of LPS-induced GRECs.

In general, the pro-oxidant and antioxidant systems in the body are in equilibrium. If a redox steady-state is imbalanced, it can lead to high free radical production. The large accumulation of free radical can induce unsaturated fatty acids to produce MDA in cells, and MDA promotes cell death by disintegrating cells. Antioxidative enzymes can scavenge free radicals and maintain a redox steady-state in the body. An increased production of ROS or a decreased defense capability of cellular antioxidant can cause oxidative stress. ROS produced in the process of oxidative metabolism could initiate the inflammatory process resulting in synthesis and secretion of proinflammatory cytokines. Furthermore, excessive ROS accumulation can cause irreversible damage to cells, which lead to cell death by the necrotic and apoptotic processes [18]. Previous studies showed that LPS can reduce the activity of antioxidative enzymes including SOD, CAT, and GSH-PX and enhance ROS and proinflammatory cytokine (IL-6, TNF-α, and IL-1β) production in cells [10,12]. The results of our study agreed with those studies. Our research findings indicate that LPS could trigger oxidative stress, leading to cell apoptosis. The chemical structure of rutin contains phenolic rings and free hydroxyl groups that can donate electrons to reactive free radicals to form more stable species and quench the free radical chain reaction [19]. Liu et al [13] and Paudel et al [14] found that LPS induced reactive ROS production of cells was significantly reduced by rutin. In addition, rutin could reverse decreased activities of antioxidative enzymes such as SOD, CAT, GSH-PX, and heme oxygenase-1 induced by LPS and improve the stressed condition by increasing the levels of GSH-PX, SOD, and decreasing MDA level [20,21]. Our study found the similar results. A cytosolic enzyme, LDH has been used as a sensitive index for assessing the degree of cell injury. Yang et al [22] reported that rutin reduced LDH levels in hypoxia/reoxygenation-induced H9c2 cells, and our results are consistent with that. Therefore, rutin could protect GRECs against damage by strongly scavenging free radical and inhibiting of lipid peroxidation.

Rutin plays a role in inhibiting the inflammatory response. According to the results of Su et al [23], rutin can decrease the protein and mRNA levels of TNF-α, IL-1β, and IL-6 in mammary tissues of mastitis model induced by LPS. Previous studies displayed rutin can suppress the production of TNF-α in LPS-induced human umbilical vein endothelial cells and decrease *IL-6* and *TNF-α* gene expression in mouse muscle cells induced by LPS, which were similar with us [13,24]. Chemokines are a class of cytokines that regulate infiltrating cell migration and adhesion to an inflamed lesion. Based on the arrangement of the amino terminal cysteine, chemokines can be divided into four subgroups: C, CC, CXC, and CX3C. CCL5 facilitates the inflammatory responses and induces T cell subset adhesion and migration in immune responses. Known as granulocyte chemotactic protein 2, CXCL6 can be upregulated after stimulation by inflammatory factors. Proinflammatory cytokines, such as TNF-α and IL-1 can induce the expression of CXCL6. Also termed IL-8, CXCL8 is a proinflammatory chemokine rapidly induced by proinflammatory cytokines such as TNF-α and IL-1, bacterial or viral products, and cellular stress. The upregulation of *CCL5*, *CXCL6*, and *CXCL8* in the LPS-induced GRECs is consistent with the results of Bulgari et al [11] and Hu et al [15]. Here, rutin reduced mRNA and protein levels of CXCL6 and CXCL8 in LPS-induced GRECs, which may be related to the reduced TNF-α levels. Therefore, rutin could attenuate LPS-induced inflammatory injury in GRECs by inhibiting the production of proinflammatory cytokines, such as TNF-α.

Toll-like receptors (TLRs) are pattern recognition receptors (PRRs) that can recognize pathogen-associated molecular patterns (PAMPs) and activate signaling pathways dependent on the adaptors MyD88 or TRIF, and then activate NF-κB and induce the release of proinflammatory cytokines, chemokines, and type I interferon (IFN). IFN regulatory factor 3 (IRF3) is the most important transcription regulatory factor of type I IFN expression and is activated in the MyD88-independent signaling pathway by TLR4. Hu et al [15] found that LPS can upregulate expression of *TLR2* and *NF-κB* in GRECs, but can not affect relative expression of *MyD88*, *IRF3*, *IFIT3*, and *TOLLIP*. In addition, LPS leads to increase in expression of *TLR4* and *NF-κB* in goat mammary epithelial cells [11]. Our study found the similar results. According to the results of previous studies, rutin downregulated the expression levels of TLR4 and NF-κB, and inhibited the phosphorylation of NF-κB in LPS-induced cells and tissues [13,20,25]. Furthermore, rutin mitigated inflammatory reaction in cells and tissues by inhibiting the protein or mRNA expressions of TLR2, TLR4, and IRF3 [26], and our results are consistent with these findings. IκBα can inhibit the activation of NF-κB, and then inhibited the transcriptional activation of genes of proinflammatory factor. The results of our study showed rutin might inhibit the activation of NF-κB by increasing the level of IκBα. Hence, rutin might reduce the expression of proinflammatory chemokines (TNF-α) and chemokines (CXCL6, CXCL8) in LPS-induced GRECs by inhibiting the TLR/NF-κB signaling pathway. A type I IFN-stimulated gene, *IFIT3* has antitumor activity. We demonstrated rutin did not affect *IFIT3* expression. This indicated that rutin might not affect the IFN secretion in cells. TOLLIP can regulate the inflammatory reaction by suppressing proinflammatory cytokines and improving anti-inflammatory secretion in cells. However, *TOLLIP* expression has no significant difference in all treatment groups. Thus, rutin could not suppresses proinflammatory cytokines in GRECs by regulating TOLLIP expression.

TEER is an important indicator which evaluate the permeability and integrity of the epithelial barrier. The higher the value of TEER, the lower the permeability of epithelial barrier. Hence, rutin can reduce the permeability of GRECs according to the result of our study. TJs, such as the Claudin, Occludin, and ZO family members, are the most important structure of the rumen epithelium barrier, regulating nutrient absorption in the epithelium and preventing microbe and toxin entry from the rumen into blood. SARA can elevate levels of LPS, high acidity, and hyperosmolality and impair ruminal epithelial barrier function by decreasing the expression of TJ proteins (Claudin-1, Occludin, ZO-1) and increasing rumen epithelium permeability [27]. However, it has also been reported that an increase of SARA-induced LPS can decrease the mRNA and protein expression of ZO-1 and increase the mRNA and protein expression of Claudin-1 [28]. This study had similar results with them. Our results confirmed that SARA induced ruminal epithelial barrier injury possibly related to the LPS-decreased TJ proteins. Previous study found that autophagy can increase the protein expression and stability of Claudin 1. Furthermore, the increased level of Claudin 1 stimulated autophagy by decreasing the level of the autophagy substrate under autophagy-inducing conditions [29]. The increased expression of Claudin 1 in LPS-induced GRECs might be relation to autophagy of cells in this study. IL-1β can inhibite the activity of transcriptional promoters of Occludin protein, leading to decrease the mRNA expression of Occludin, and then destroy the effects of TJ. Compared to the control, the expression of Occludin was slightly decreased in LPS-induced GRECs, might be relation to an increased level of IL-1β. Flavonoids, such as quercetin, morin, naringenin, enhance barrier functions of intestinal epithelial cells stimulated with high glucose by increasing Claudin-1, Occludin, and ZO-1 expression [30]. We also found rutin can enhance the expression levels of Claudin-1 and ZO-1 in LPS-induced GRECs. This indicated that rutin could relieve ruminal epithelial barrier function injury in GRECs induced by LPS. Therefore, rutin may protect the ruminal epithelial barrier against SARA, which nevertheless requires verification in the future.

## CONCLUSION

LPS decreased the activity and increased apoptosis of GRECs and caused barrier injury in GRECs by promoting oxidation stress and the inflammatory response. Rutin alleviated the barrier injury of LPS-induced GRECs by enhancing oxidation resistance and anti-inflammation, which may be connected with inhibition of the TLR/NF-κB signaling pathway.

## Figures and Tables

**Table 1:** Real-time quantitative polymerase chain reaction primers

Gene	Accession number	Primer sequence (5′-3′)	Product size (bp)
*IL-1β*	XM_013967700.2	F:GAAGAGCTGCACCCAACA R:CAGGTCATCATCACGGAAG	172
*IL-6*	NM_001285640	F:AGATATACCTGGACTTCCT R:TGTTCTGATACTGCTCTG	80
*TNF-α*	NM_001286442	F:TGGTTCAGACACTCAGGT R:CGCTGATGTTGGCTACAA	75
*CCL5*	XM_005693201	F:GTCTGCCTCCCCATATGCCTC R:CTCTCGCACCCACTTCTTCTC	187
*CXCL6*	XM_005681937	F:CCAAGGTGGAAGTGGTAGCC R:CTGGGCAATTCTTCCAACGC	149
*CXCL8*	XM_005681749	F:TGTGTGAAGCTGCAGTTCTGT R:TGGGGTCTAAGCACACCTCT	186
*Claudin-1*	XM_005675123	F:CACCCTTGGCATGAAGTGTA R:AGCCAATGAAGAGAGCCTGA	216
*Occludin*	XM_018065677.1	F:GTTCGACCAATGCTCTCTCAG R:CAGCTCCCATTAAGGTTCCA	200
*IFIT3*	XM_005698196	F:AAATTCTGAGGCAGGCCGTT R:TTTCCCAGAGCCTCGACAAC	127
*MyD88*	XM_013973392	F:TTGAGAAGAGGTGCCGTCG R:CAGACAGTGATGAAGCGCAG	187
*IRF3*	XM_013971473	F:TTGTGAACTCAGGGGTCAGG R:TGGGCTCAAGTCCATGTCAC	125
*NF-κB*	XM_005681365	F:CTGGAAGCACGAATGACAGA R:GCTGTAAACATGAGCCGTACC	197
*TLR2*	NM_001285603	F:TTGACAAGAAGGCCATCCCC R:AGAACGCTTCCTGCTGAGTC	105
*TLR4*	NM_001285574	F:TTCAACCGTATCACGGCCTC R:TGACCCACTGCAGGAAACTC	127
*TOLLIP*	XM_013976999	F:CGACGTAGGCTTAGCGTGAA R:CTGGTCTCACGCATCTACCG	142
*GAPDH*	XM_005680968.2	F:CAAAGTGGACATCGTTGCCA R:TGGAAGATGGTGATGGCCTT	197

*IL-1β*, interleukin-1β; *TNF-α*, tumor necrosis factor-α; *CCL5*, C-C motif chemokine ligand 5; *CXCL6*, C-X-C motif chemokine ligand 6; *IFIT3*, interferon induced protein with tetratricopeptide repeats 3; *MyD88*, myeloid differentiation primary response gene 88; *IRF3*, interferon (IFN) regulatory factor 3; *NF-κB*, nuclear factor κB; TLR2, toll-like receptor 2; *TOLLIP*, toll-interacting protein; *GAPDH*, glyceraldehyde-3-phosphate dehydrogenase.

**Table 2:** Effects of rutin on the antioxidative parameters of LPS-induced GRECs

Parameters	Treatment groups		

	Con	L	L+R
Lactate dehydrogenase (U/L)	2.49^B^±0.34	3.88^A^±0.50	1.14^C^±0.07
Superoxide dismutase (U/mL)	14.16^A^±0.20	8.99^C^±0.45	10.94^B^±0.30
Glutathione peroxidase (U/mL)	44.88^A^±1.38	26.71^B^±0.25	28.21^B^±0.68
Catalase (U/mL)	6.49^A^±0.11	4.30^C^±0.03	4.88^B^±0.11
Reactive oxygen species (Fluorescence strength/mL)	22.64^C^±0.74	44.57^A^±0.51	38.30^B^±0.26
Malondialdehyde (nmol/mL)	3.15^C^±0.04	4.64^A^±0.03	4.36^B^±0.05

LPS, lipopolysaccharide; GRECs, goat rumen epithelial cells.

1) Con, control group; L, LPS group; L+R, LPS+rutin group.

Means with capital letters in the same row indicate significantly different (p<0.01).

**Table 3:** Effects of rutin on the relative cytokine expression in LPS-induced GRECs

Item	Treatment groups^1)^		

	Con	L	L+R
*IL-6*	1.00^b^±0.02	2.17^a^±0.23	1.88^ab^±0.39
*TNF-α*	1.01^C^±0.07	5.75^A^±0.33	2.57^B^±0.04
*IL-1β*	1.01^B^±0.07	2.33^A^±0.10	2.37^A^±0.12
*CCL5*	1.02^B^±0.13	5.55^A^±0.32	5.14^A^±0.73
*CXCL6*	1.00^C^±0.01	9.42^A^±1.24	7.31^B^±0.04
*CXCL8*	1.00^C^±0.03	16.80^A^±1.34	14.68^B^±0.21

LPS, lipopolysaccharide; GRECs, goat rumen epithelial cells; IL-6, interleukin-6; TNF-α, tumor necrosis factor-α; IL-1β, interleukin-1β; CCL5, C-C motif chemokine ligand 5; CXCL6, C-X-C motif chemokine ligand 6.

1) Con, control group; L, LPS group; L+R: LPS+rutin group.

Means with lowercase letters (p<0.05) and capital letters (p<0.01) in the same row indicate significantly different.

**Table 4:** Effects of rutin on the relative expression of regulatory factor genes in LPS-induced GRECs

Item	Treatment groups^1)^		

	Con	L	L+R
*TLR2*	1.06^C^±0.17	3.80^A^±0.43	2.45^B^±0.08
*TLR4*	1.01^b^±0.06	1.37^a^±0.14	0.76^b^±0.04
*NF-κB*	1.01^C^±0.05	2.63^A^±0.22	1.85^B^±0.09
*MyD88*	1.01^b^±0.05	1.48^ab^±0.30	2.09^a^±0.33
*IFIT3*	1.01±0.06	1.12±0.07	1.03±0.15
*IRF3*	1.01^a^±0.06	1.05^a^±0.02	0.81^b^±0.02
*TOLLIP*	1.01±0.05	0.91±0.11	0.79±0.02

LPS, lipopolysaccharide; GRECs, goat rumen epithelial cells; TLR2, toll-like receptor 2; NF-κB, nuclear factor κB; MyD88, differentiation primary response gene 88?; IFIT3, interferon induced protein with tetratricopeptide repeats 3; IRF3, interferon (IFN) regulatory factor 3; TOLLIP, toll-interacting protein.

1) Con: control group; L, LPS group; L+R, LPS+Rutin group.

Means with lowercase letters (p<0.05) and capital letters (p<0.01) in the same row indicate significantly different.

**Table 5:** Effects of rutin on protein contents of cytokines in LPS-induced GRECs

Item	Treatment groups^1)^		

	Con	L	L+R
TLR2 (ng/mg)	11.32±0.68	11.92±0.42	11.47±0.44
TLR4 (ng/mg)	6.22^b^±0.16	6.67^a^±0.11	6.21^b^±0.10
NF-κB (ng/mg)	636.08^b^±5.32	718.07^a^±24.84	695.02^ab^±24.92
IκBα (ng/g)	877.52±21.83	854.76±42.34	936.44±21.25
IL-6 (ng/L)	175.57^a^±5.02	184.83^a^±2.00	152.24^b^±3.79
TNF-α (ng/L)	1,273.78^ab^±57.28	1,425.37^a^±48.15	1,201.84^b^±33.19
IL-1β (ng/L)	123.81^b^±4.31	136.38^a^±3.31	134.07^ab^±2.70
CXCL6 (ng/L)	241.81±12.10	275.21±10.05	239.95±10.90
CXCL8 (ng/L)	98.31^b^±2.81	108.02^a^±1.80	94.59^b^±2.37

LPS, lipopolysaccharide; GRECs, goat rumen epithelial cells; TLR2, toll-like receptor 2; NF-κB, nuclear factor κB; IκBα, inhibitor of nuclear factor kappa-B alpha; IL-6, interleukin-6; TNF-α, tumor necrosis factor-α; IL-1β, interleukin-1β; CXCL6, C-X-C motif chemokine ligand 6.

1) Con, control group; L, LPS group; L+R, LPS+rutin group.

Means with lowercase letters in the same row indicate significantly different (p<0.05).
